# Supplementary material for: Pyruvate Dehydrogenase Kinase 1 inhibition mediated oxidative phosphorylation enhancement in cartilage promotes osteoarthritis progression
Source: BMC Musculoskelet Disord. 2023 Jul 20;24:597. doi: 10.1186/s12891-023-06585-6 (PMC10357736; doi:10.1186/s12891-023-06585-6)
Supplement: Supplementary file 1 — Additional file 1. [file 12891_2023_6585_MOESM1_ESM.zip › MF.Gsea.1653627316242/GOMF_AMIDE_TRANSMEMBRANE_TRANSPORTER_ACTIVITY.html]

Details for gene set GOMF\_AMIDE\_TRANSMEMBRANE\_TRANSPORTER\_ACTIVITY[GSEA]

|  || Dataset | 55457\_collapsed\_to\_symbols.cls.cls #OA\_versus\_normal.cls.cls #OA\_versus\_normal\_repos |
| Phenotype | cls.cls#OA\_versus\_normal\_repos |
| Upregulated in class | normal |
| GeneSet | GOMF\_AMIDE\_TRANSMEMBRANE\_TRANSPORTER\_ACTIVITY |
| Enrichment Score (ES) | -0.44106364 |
| Normalized Enrichment Score (NES) | -1.5130538 |
| Nominal p-value | 0.03588907 |
| FDR q-value | 0.17465349 |
| FWER p-Value | 1.0 |
Table: GSEA Results Summary

  

Fig 1: Enrichment plot: GOMF\_AMIDE\_TRANSMEMBRANE\_TRANSPORTER\_ACTIVITY      
 Profile of the Running ES Score & Positions of GeneSet Members on the Rank Ordered List

  

| SYMBOL | TITLE | RANK IN GENE LIST | RANK METRIC SCORE | RUNNING ES | CORE ENRICHMENT || 1 | SLC15A3 | solute carrier family 15 member 3 [Source:HGNC Symbol;Acc:HGNC:18068] | 2612 | 0.290 | -0.1626 | No |
| 2 | SLC33A1 | solute carrier family 33 member 1 [Source:HGNC Symbol;Acc:HGNC:95] | 3320 | 0.219 | -0.1871 | No |
| 3 | ABCC4 | ATP binding cassette subfamily C member 4 [Source:HGNC Symbol;Acc:HGNC:55] | 3344 | 0.217 | -0.1587 | No |
| 4 | AQP3 | aquaporin 3 (Gill blood group) [Source:HGNC Symbol;Acc:HGNC:636] | 3805 | 0.176 | -0.1700 | No |
| 5 | SLC14A1 | solute carrier family 14 member 1 (Kidd blood group) [Source:HGNC Symbol;Acc:HGNC:10918] | 4066 | 0.150 | -0.1694 | No |
| 6 | TAPBP | TAP binding protein [Source:HGNC Symbol;Acc:HGNC:11566] | 4527 | 0.111 | -0.1896 | No |
| 7 | PDPN | podoplanin [Source:HGNC Symbol;Acc:HGNC:29602] | 5594 | 0.024 | -0.2691 | No |
| 8 | SLC38A7 | solute carrier family 38 member 7 [Source:HGNC Symbol;Acc:HGNC:25582] | 5916 | 0.001 | -0.2939 | No |
| 9 | ABCB9 | ATP binding cassette subfamily B member 9 [Source:HGNC Symbol;Acc:HGNC:50] | 6442 | -0.038 | -0.3293 | No |
| 10 | SLC15A1 | solute carrier family 15 member 1 [Source:HGNC Symbol;Acc:HGNC:10920] | 6515 | -0.043 | -0.3289 | No |
| 11 | TAP1 | "transporter 1, ATP binding cassette subfamily B member [Source:HGNC Symbol;Acc:HGNC:43]" | 6633 | -0.052 | -0.3307 | No |
| 12 | SLC15A2 | solute carrier family 15 member 2 [Source:HGNC Symbol;Acc:HGNC:10921] | 6704 | -0.056 | -0.3284 | No |
| 13 | ABCG2 | ATP binding cassette subfamily G member 2 (Junior blood group) [Source:HGNC Symbol;Acc:HGNC:74] | 7473 | -0.116 | -0.3719 | No |
| 14 | AQP9 | aquaporin 9 [Source:HGNC Symbol;Acc:HGNC:643] | 7933 | -0.150 | -0.3868 | No |
| 15 | AQP7 | aquaporin 7 [Source:HGNC Symbol;Acc:HGNC:640] | 8136 | -0.165 | -0.3795 | No |
| 16 | ABCD1 | ATP binding cassette subfamily D member 1 [Source:HGNC Symbol;Acc:HGNC:61] | 8930 | -0.226 | -0.4096 | Yes |
| 17 | ABCC5 | ATP binding cassette subfamily C member 5 [Source:HGNC Symbol;Acc:HGNC:56] | 9066 | -0.237 | -0.3871 | Yes |
| 18 | SLC25A32 | solute carrier family 25 member 32 [Source:HGNC Symbol;Acc:HGNC:29683] | 9170 | -0.246 | -0.3609 | Yes |
| 19 | GJA1 | gap junction protein alpha 1 [Source:HGNC Symbol;Acc:HGNC:4274] | 9325 | -0.258 | -0.3371 | Yes |
| 20 | SLC14A2 | solute carrier family 14 member 2 [Source:HGNC Symbol;Acc:HGNC:10919] | 9616 | -0.284 | -0.3201 | Yes |
| 21 | SLC19A1 | solute carrier family 19 member 1 [Source:HGNC Symbol;Acc:HGNC:10937] | 9931 | -0.315 | -0.3007 | Yes |
| 22 | SLC13A3 | solute carrier family 13 member 3 [Source:HGNC Symbol;Acc:HGNC:14430] | 10096 | -0.330 | -0.2675 | Yes |
| 23 | SLC47A1 | solute carrier family 47 member 1 [Source:HGNC Symbol;Acc:HGNC:25588] | 10423 | -0.363 | -0.2423 | Yes |
| 24 | SLC38A3 | solute carrier family 38 member 3 [Source:HGNC Symbol;Acc:HGNC:18044] | 10443 | -0.366 | -0.1928 | Yes |
| 25 | CDH17 | cadherin 17 [Source:HGNC Symbol;Acc:HGNC:1756] | 10667 | -0.390 | -0.1559 | Yes |
| 26 | ABCC1 | ATP binding cassette subfamily C member 1 [Source:HGNC Symbol;Acc:HGNC:51] | 11462 | -0.497 | -0.1485 | Yes |
| 27 | TAP2 | "transporter 2, ATP binding cassette subfamily B member [Source:HGNC Symbol;Acc:HGNC:44]" | 11616 | -0.519 | -0.0882 | Yes |
| 28 | SLC5A6 | solute carrier family 5 member 6 [Source:HGNC Symbol;Acc:HGNC:11041] | 11809 | -0.551 | -0.0264 | Yes |
| 29 | SLC19A2 | solute carrier family 19 member 2 [Source:HGNC Symbol;Acc:HGNC:10938] | 12600 | -0.798 | 0.0233 | Yes |
Table: GSEA details [plain text format]

  

Fig 2: GOMF\_AMIDE\_TRANSMEMBRANE\_TRANSPORTER\_ACTIVITY      
 Blue-Pink O' Gram in the Space of the Analyzed GeneSet

  

Fig 3: GOMF\_AMIDE\_TRANSMEMBRANE\_TRANSPORTER\_ACTIVITY: Random ES distribution      
 Gene set null distribution of ES for **GOMF\_AMIDE\_TRANSMEMBRANE\_TRANSPORTER\_ACTIVITY**
